# Anatomical Features in Inguinal-Pubic-Adductor Area That May Contribute to Gender Difference in Susceptibility to Groin Pain Syndrome

**DOI:** 10.3390/jpm14080860

**Published:** 2024-08-14

**Authors:** Gian Nicola Bisciotti, Andrea Bisciotti, Alessio Auci, Alessandro Bisciotti, Piero Volpi

**Affiliations:** 1 Kinemove Rehabilitation Centers, 54027 Pontremoli, Italy; alessandrobisciotti@icloud.com; 2 IRCCS Humanitas Research Hospital, 20089 Milan, Italy; andrea.bisciotti@humanitas.it (A.B.); piero.volpi@humanitas.it (P.V.); 3 Dipartimento delle Diagnostiche, Azienda USL Toscana Nord Ovest, 56121 Massa, Italy; alessio.auci@uslnordoves.toscana.it

**Keywords:** pubic anatomy, inguinal anatomy, pelvic anatomy, hip joint anatomy, sexual apparatus

## Abstract

Groin pain syndrome (GPS) is often a diagnostic challenge for sport physicians. Despite this diagnostic difficulty, the incidence of GPS in athletes is relatively high, afflicting 10–20% of the total sports population. In the literature, a certain number of studies demonstrate an important gender-based difference in the incidence of GPS in both sexes, with a ratio of female:male athletes clearly in favor of the female gender being relatively less prone to GPS. Indeed, some anatomical differences between the two sexes seem to represent a protective factor against the onset of GPS in women, although the current literature still needs to clarify the validity of these findings. It is the aim of this systematic review to examine all the anatomical differences between men and women that may be responsible for the difference in the onset of GPS in the two sexes.

## 1. Introduction

Groin Pain Syndrome (GPS) is on the rise and calls for further understanding [1,2,3,4]. Defined as “any clinical symptom reported by the patient, located at the inguinal-pubic-adductor area, affecting sports activities and/or interfering with Activities of Daily Living (ADL) and requiring medical attention” by the “Groin Pain Syndrome Italian Consensus Conference on terminology, clinical evaluation and imaging assessment in groin pain in athletes”, GPS presents a multifactorial etiopathogenesis [3,4]. Diagnosis is often challenging due to the anatomical and biomechanical complexity of the groin region, and a full understanding of this syndrome is complicated by the sheer number of clinical conditions that can trigger this condition. Therefore, a multidisciplinary approach is best taken for a correct diagnosis and for securing the most appropriate conservative and surgical treatments.

The 2016 GPS Italian Consensus Conference approved an initial classification of this syndrome based on the pathogenesis of the condition and the symptoms experienced [3].

GPS of traumatic origin: here, pain is triggered by an acute trauma of any sort, and clinical, medical, and imaging records are available for consultation to back this diagnosis.

GPS due to functional overload: this case is characterized by a known cause or by an insidious and progressive onset unaccompanied by acute trauma.

Chronic or long-standing GPS (LSGPS): here, the patient suffers from symptoms that are recalcitrant to any conservative therapy for at least 12 weeks.

This classification system was recently revised during The Groin Pain Syndrome Italian Consensus Conference update of 2023 [4]: the previously identified 11 nosological categories referring to GPS are now 12, and these currently account for 67 clinical situations as opposed to 63.

GPS is on the rise in many sports such as football, rugby, soccer, ice hockey, and handball [1,2,3,4]: in soccer, for example, 10–18% of all time-loss injuries can already be attributed to GPS [1]. Athletes who practice these sports employ vigorous and complicated movements such as kicking, changes in direction, and cutting maneuvers, which, coupled with high activity loads and short recovery periods between matches, contribute to the growing incidence of GPS. Moreover, several recent studies [5,6,7,8,9] demonstrate an important difference in the incidence of GPS in male and female athletes. Whereas these findings may partially be explained by the different extent and intensity of training loads and/or the different match workloads, a gender-based component is becoming ever more apparent. Indeed, the risk of developing GPS is higher in male athletes than it is in female athletes and may very well depend on anatomical gender-related differences regarding, in particular, the pubis symphysis, inguinal canal, pelvis and hip joint morphology, and the sexual apparatus.

The aim of this systematic review is to analyze these factors as possible causes of the difference with which GPS occurs in male and female athletes.

## 2. Materials and Methods

### 2.1. Aim of the Current Systematic Review

This systematic review was conducted in accordance with the PRISMA (Preferred Reporting Items for Systematic Reviews and MetaAnalysis) guidelines [10]. The protocol of this study is in the registration process at the PROSPERO register for systematic reviews.

### 2.2. Data Extraction and Quality Assessment

**Figure 1:**
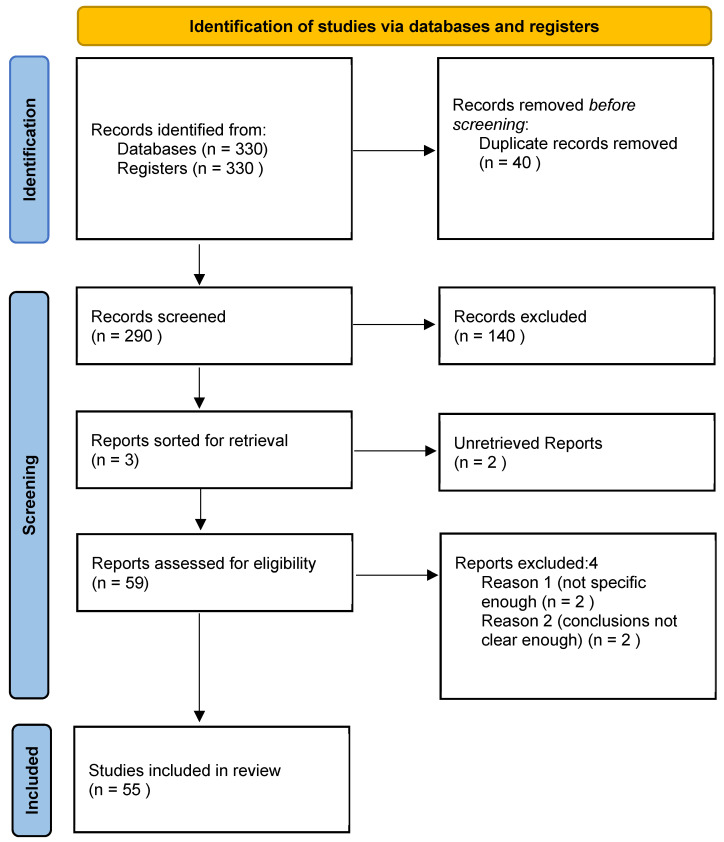
The PRISMA flow diagram of the study search and selection procedure.

The PubMed/MEDLINE, Scopus, ISI. Cochrane Database of Systematic Reviews and PEDro databases were consulted for systematic reviews on the role of gender differences in the onset of GPS in order to guarantee the originality of this systematic review. After this initial verification, three authors (G.N.B., A.B., and A.A.) independently screened the literature using a string of keywords: “groin pain syndrome”, “pubalgia”, “athletic pubalgia”, “sport hernia”, “inguinal hernia”, “femoral hernia“, “femoroacetabular impingement”, “hip dysplasia”, “hip joint anatomy”, “pelvic anatomy”, “inguinal anatomy”, “sexual apparatus”, fittingly connected by Boolean operators. When appropriate, medical subject headings (MeSH) and wild-card options were used. Furthermore, target journals were reviewed in order to collate the maximum number of relevant articles. This phase of research spanned the period 20 June 2024–30 June 2024. Neither data restriction nor language limitation were applied. “Grey literature”, i.e., conference accounts, abstracts, thesis, and unpublished reports, was not taken into account. Cross-references from the selected articles were screened to verify their possible relevance. All double citations were removed. For each article, the relevant information was extracted and recorded on an ad hoc Excel spreadsheet. The PRISMA flow diagram of the study search and selection procedure is shown in Figure 1. The results of quality assessment of each individual study considered is shown in Table 1 and was performed in agreement with the Joanna Briggs Institute quantitative critical appraisal tools [11].

### 2.3. Search Strategy Items Details

Databases consulted: PubMed/MEDLINE, Scopus, ISI. Cochrane Database of Systematic Reviews, and PEDro.

Search string: (groin pain syndrome OR pubalgia OR athletic pubalgia) AND (inguinal hernia OR femoral hernia) AND (femoroacetabular impingement OR hip dysplasia OR hip joint anatomy) AND (pelvic anatomy OR inguinal anatomy) AND (sexual apparatus). The inclusion and exclusion criteria were based on the PICO tool [12].

### 2.4. Inclusion Criteria

**P**: randomized controlled trials, case series studies, cross sectional studies, cohort studies, systematic review, narrative review, prospective studies, retrospective studies, comparative studies, multicenter studies, and editorials focused on gender difference in GPS onset. 

**I**: anatomical and clinical studies focused on gender differences in GPS pathogenesis.

**C**: comparison between the anatomical predispositions for GPS onset in male and female populations.

**O**: outcome in terms of gender difference.

### 2.5. Exclusion Criteria

**P**: randomized controlled trials, case series studies, cross sectional studies, cohort studies, systematic review, narrative review, prospective studies, retrospective studies, comparative studies, multicenter studies, and editorials focused on GPS onset without considering gender difference.

**I**: anatomical and clinical studies that did not take into account the gender difference in GPS pathogenesis.

**C**: studies in which the comparison between the anatomical predispositions for GPS onset in male and female populations is missing.

**O**: lack of outcome concerning gender difference.

### 2.6. Statistical Analysis

Since this systematic review is purely descriptive in nature, no quantitative statistical analysis was performed.

### 2.7. Results of Systematic Review

Fifty-five of the original 320 articles screened were included and summarized in this systematic review (Table 1). Each study was checked to identify any potential conflicts of interest.

### 2.8. Study Design

The studies chosen presented the following:

13 retrospective cohort studies [5,9,13,14,15,16,17,18,19,20,21,22,23],

12 prospective cohort studies [1,2,7,8,24,25,26,27,28,29,30,31],

11 narrative reviews [32,33,34,35,36,37,38,39,40,41,42],

8 systematic reviews [3,4,6,43,44,45,46,47],

3 comparative studies [48,49,50],

2 case series [51,52],

2 cross sectional studies [53,54],

2 cohort studies [55,56], and

2 editorials [57,58].

These studies were then subdivided into 5 groups based on the anatomical region they dealt with: 

(1) Pubic anatomy.

(2) Inguinal anatomy.

(3) Pelvic anatomy.

(4) Hip joint anatomy.

(5) Sexual apparatus.

**Table 1:** Study design, level of evidence, JBI score, risk of bias, and synthesis of the study concerning each considered study. Risk of bias = low IF ≥ 75% of the requested criteria were satisfied by the studies; risk of bias = moderate IF 60–74% of the requested criteria were satisfied by the studies; risk of bias = high IF < 60% of the requested criteria were satisfied by the studies [11].

Reference	Study Design	Level of Evidence	JBI Score	Risk of Bias	Synthesis of the Study
Bisciotti et al., 2016 [3]	Systematic review	I	90/100	Low	Groin Pain Syndrome Italian Consensus Conference on terminology, clinical evaluation and imaging assessment in groin pain in athlete
Bisciotti et al., 2023 [4]	Systematic review	I	90/100	Low	Groin Pain Syndrome Italian Consensus Conference update 2023.
Orchard, 2015 [6]	Systematic review	I	78/100	Low	Risk factors for groin pain syndrome in elite team sport
Becker et al., 2010 [43]	Systematic review	I	66/100	Moderate	The anatomical and physiological factors of the adult human pubic symphysis
HerniaSurge Group, 2018 [44]	Systematic review	I	90/100	Low	International guidelines for groin hernia management
Zini et al., 2023 [45]	Systematic review	I	90/100	Low	Italian Consensus Conference on FAI syndrome in athletes
Fairley et al., 2016 [46]	Systematic review	I	81/100	Low	Management for femoroacetabular impingement
Simons et al., 2009 [47]	Systematic review	I	89/100	Low	European Hernia Society guidelines on the treatment of inguinal hernia in adult patients.
Agricola et al., 2014 [55]	Cohort study	III	75/100	Low	Physiological and anatomical aspects of cam-deformity during skeletal maturation
Rosendahl et al., 1996 [56]	Cohort study	III	65/100	Moderate	Physiological and anatomical aspects of hip dysplasia
Hölmich, 2007 [1]	Prospective study	IV	40/100	High	Prospective study of 207 athletic patients affected by long-standing groin pain syndrome
Mosler et al., 2018 [2]	Prospective study	IV	50/100	High	Epidemiology of time-loss groin injuries in a professional football league
Bisciotti et al., 2021 [7]	Prospective study	IV	89/100	Low	Multidisciplinary assessment of 320 athletes with long-standing groin pain syndrome in keeping with the Italian consensus agreement
Bisciotti et al., 2022 [8]	Prospective study	IV	89/100	Low	Multidisciplinary assessment of long-standing groin pain syndrome in athletic women in keeping with the Italian Consensus Agreement
Hachisuka, 2003 [24]	Prospective study	IV	62/100	Moderate	Femoral hernia in female population anatomical, clinical and surgical description
Glassow F, 1973 [25]	Prospective study	IV	66/100	Moderate	Anatomical description of the posterior wall of the inguinal canal in women
Amid, 2005 [26]	Prospective study	IV	80/100	Low	Etiology and repair of inguinal hernia
Satpathy et al., 2015 [27]	Prospective study	IV	77/100	Low	Biomechanical study of the hip contact stress and femoral neck retroversion and their implication in femoroacetabular impingement
Nepple et al. 2014 [28]	Prospective study	IV	71/100	Moderate	The different characteristics of femoroacetabular impingement in female and male population
Koch et al., 2005 [29]	Prospective study	IV	66/100	Moderate	Prospective evaluation of 6895 groin hernia repairs in women
Herrington, 1975 [30]	Prospective study	IV	47/100	High	Anatomical and clinical description of Occult inguinal hernia in women.
Byrne et al., 2017 [31]	Prospective study	IV	65/100	Moderate	Correlation between MRI- findings and outcome after fluoroscopy-guided injection of steroid and local anesthetic in a cohort of patients affected by GPS
Zoga et al. 2008 [5]	Retrospective study	IV	80/100	Low	GPS MRI findings
Hynes et al., 2022 [9]	Retrospective study	IV	73/100	Moderate	Patterns of injury at MRI and gender differences
Schilders et al., 2021 [13]	Retrospective study	IV	70/100	Moderate	Descriptive MRI findings in 145 athletes of both sex affected by GPS
Schilders, 2000 [14]	Retrospective study	IV	53/100	High	Descriptive anatomical findings in athletes of both sex affected by GPS
Lytle, 1979 [15]	Retrospective study	IV	61/100	Moderate	Differences in inguinal anatomy between male and female.
Spangen et al., 1998 [16]	Retrospective study	IV	60/100	Moderate	Non-palpable inguinal hernia in the female population
López-Cano M et al., 2005 [17]	Retrospective study	IV	65/100	Moderate	Anthropometric characteristics of the pubic arch and the function of the defense mechanisms against hernia formation.
Mitrousias et al., 2023 [18]	Retrospective study	IV	70/100	Moderate	Anatomy and terminology of groin pain
Miller, 2018 [19]	Retrospective study	IV	78/100	Low	Inguinal anatomy
Tague, 2000 [20]	Retrospective study	IV	59/100	High	Female pelvis anatomy
Bisciotti et al., 2022 [21]	Retrospective study	IV	80/100	Low	Correlation between imaging parameters, sport activity, chondral damage, and femoroacetabular impingement
Di Pietto et al., 2017 [22]	Retrospective study	IV	67/100	Moderate	Postoperative imaging in arthroscopic hip surgery in both sex
Kark and Kurzer, 2008 [23]	Retrospective study	IV	49/100	High	Anatomical description of groin hernia in women
Rosen et al., 1989 [48]	Comparative study	IV	50/100	High	The anatomical differences in the inguinal region in men and women with reference to inguinal hernia formation.
Abdalla and Mittelstaedt, 2001 [49]	Comparative study	IV	48/50	High	Anatomical description of Hessert’s triangle in the etiology of inguinal hernia
Nakahara et al., 2011 [50]	Comparative study	IV	61/50	Moderate	3D morphological study for gender differences in bony impingement of human hips
Byrd and Jones, 2011 [51]	Case series	IV	48/100	High	Arthroscopic management of femoroacetabular impingement in athletic patients
Zoland et al., 2018 [52]	Case series	IV	50/100	High	Groin pain syndrome in female athletic population
Johnson et al., 2012 [53]	Cross sectional study	IV	51/100	High	Femoroacetabular impingement in high-level youth soccer players
Agricola et al., 2012 [54]	Cross sectional study	IV	61/100	Moderate	Anatomical and physiological aspects of the development of Cam-type deformity in adolescent and young male soccer players
McMinn, 1994 [32]	Narrative review	V	Not applicable	Not applicable	Anatomical description of the pelvis in the male and female populations
Eickmeyer, 2017 [33]	Narrative review	V	Not applicable	Not applicable	Anatomical and physiological description of the pelvic floor
Gamble et al., 1986 [34]	Narrative review	V	Not applicable	Not applicable	Anatomic and pathologic considerations concerning the pubis symphysis
Bisciotti et al., 2022 [35]	Narrative review	V	Not applicable	Not applicable	Prepubic aponeurotic complex anatomical description
Thorborg., 2023 [36]	Narrative review	V	Not applicable	Not applicable	Groin pain taxonomy and anatomical description
Shakil et al., 2020 [37]	Narrative review	V	Not applicable	Not applicable	Diagnosis and management of inguinal hernias
Bou Antoun et al., 2018 [38]	Narrative review	V	Not applicable	Not applicable	Imaging of inguinal-related groin pain syndrome in athletic population
van Veenendaal et al., 2023 [39]	Narrative review	V	Not applicable	Not applicable	Treatment of chronic postoperative inguinal pain
Lozada-Martinez et al., 2022 [40]	Narrative review	V	Not applicable	Not applicable	Pre-operative factors associated with short- and long-term outcomes in the patient with inguinal hernia
Forlizzi et al., 2023 [41]	Narrative review	V	Not applicable	Not applicable	Evaluation and treatment of core muscle injury in the athletes
Packer and Safran, 2015 [42]	Narrative review	V	Not applicable	Not applicable	Etiology of primary femoroacetabular impingement
Schache et al., 2017 [57]	Editorial	V	Not applicable	Not applicable	Anatomical and morphological characteristics of groin pain syndrome in female athletes
Kaplan et al., 2019 [58].	Editorial	V	Not applicable	Not applicable	Surgical aspect of occult inguinal hernia repair

## 3. Pubic Anatomy

The ilium, ischium, pubis, sacrum, and coccyx together form the pelvis. The left and right superior rami of the pubis bones meet anteriorly along a midline to form a joint called the pubic symphysis. The specific nature of this joint permits it to distribute shear forces during ambulation and to resist tensile, shearing, and compressive forces with limited mobility. Indeed, in physiological conditions, the pubis symphysis shows a maximal rotation of 1° and a maximum shift of 2 mm [43]. The pubic symphysis is currently classified as a “secondary cartilaginous joint” [32] or a “fibrocartilaginous joint” [33]. However, with the last anatomical study on the pubic symphysis dating back to 1986 [34], a lot remains to be understood about this joint. Indeed, the lack of recent anatomical studies has not furthered our understanding of the etiopathogenesis of certain pelvic disorders that may ultimately lead to the onset of GPS.

**Figure 2:**
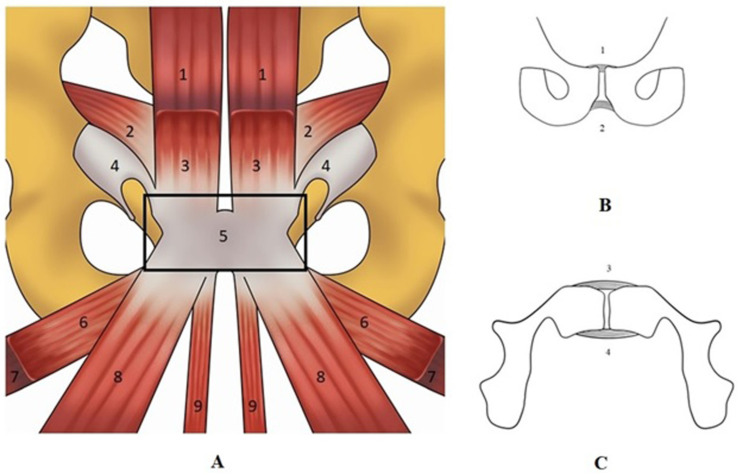
A schematic view of the tendon structure forming the prepubic aponeurotic complex (box (**A**)) and a schematic view of the pubic ligaments in coronal view (box (**B**)) and axial view (box (**C**)). The prepubic aponeurotic complex is formed by the anterior, the inferior, and the superior pubic ligaments. Legend Box (**A**): (1) Rectus abdominis; (2) Tranversus abdominis and internal oblique; (3) Piramidalis; (4) External oblique; (5) Pre-pubic aponeurotic complex.; (6) Pectineus; (7) Adductor brevis; (8) Adductor longus; (9) Gracilis. Legend box (**B**,**C**): (1) Superior pubic ligaments; (2) Inferior pubic ligament; (3) Anterior pubic ligament; (4) Posterior pubic ligament.

The prepubic aponeurotic complex (PPAC), depicted in Figure 2, is an important component of the pelvis. This PPAC is formed by the interconnection between the tendons of the adductor longus, adductor brevis, gracilis, and pectineus muscles, the aponeurosis of rectus abdominis, pyramidalis, and external oblique muscles, the articular disc, the anterior pubic periostium and by the superior (SPL), inferior (IPL) and anterior (APL) pubic ligaments, while the posterior pubic ligament (PPL) is not part of the PPAC [17,18]. PPAC lesions, both of traumatic origin and from overuse, are an important cause of GPS in athletic populations [13,35]. A study focusing on the dissection of sixteen embalmed cadavers (eight men and eight women) showed several sex-related differences in the tissues forming the PPAC [19]. Specifically, in female subjects, the medial part of the rectus abdominis muscle is inserted directly into the antero-superior part of the pubic symphysis. On the contrary, in male cadavers, the medial part of the rectus abdominis tendon continues over the anterior surface of the symphysis, blending distally with the proximal attachment of the gracilis muscle. The study reported that the tendinous extension was present bilaterally and, on average, was between 0.7 and 0.9 cm wide.

A possible hypothesis is that this recto-gracilis tendinous extension may be more exposed to higher tensile forces compared to the morphology observed in female cadavers. Thus, the male population could be more exposed to trauma and/or overuse of the PPAC portion afferent to the recto-gracilis junction. However, it is important to underline that this study was strongly limited by the restricted number of cadavers dissected; therefore, these anatomical observations need to be confirmed or contested by further studies.

## 4. Inguinal Anatomy

**Figure 3:**
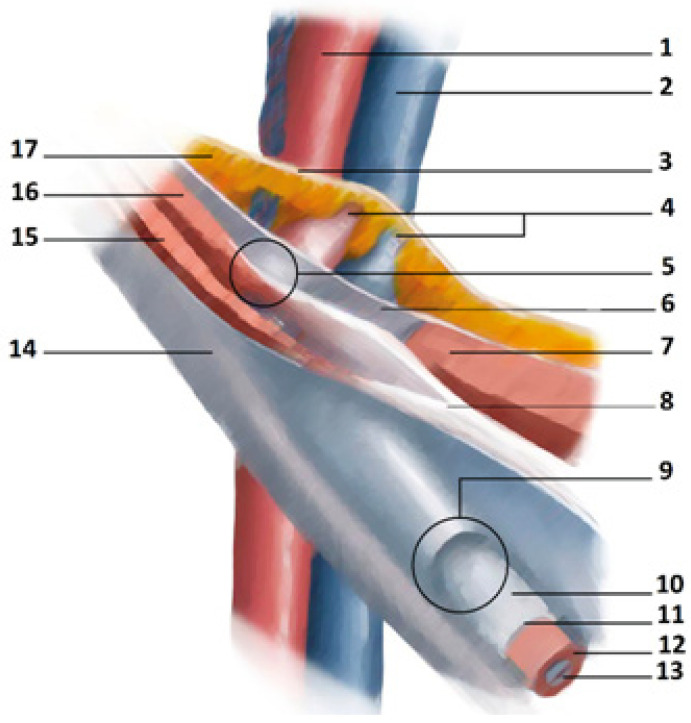
The inguinal canal: a schematic view. Legend: (1) External iliac artery. (2) External iliac vein. (3) Parietal peritenonium. (4) Inferior epigastric vessels. (5) Internal inguinal ring. (6) Transversalis fascia. (7) Rectus abdominis muscle. (8) Conjoint tendon. (9) External inguinal ring. (10) Spermatic cord. (11) External spermatic fascia. (12) Cremasteric muscle and fascia. (13) Internal spermatic fascia. (14) External oblique muscle aponeurosis. (15) Internal oblique muscle. (16) Transversus oblique muscle. (17) Extra-peritoneal tissue.

**Figure 4:**
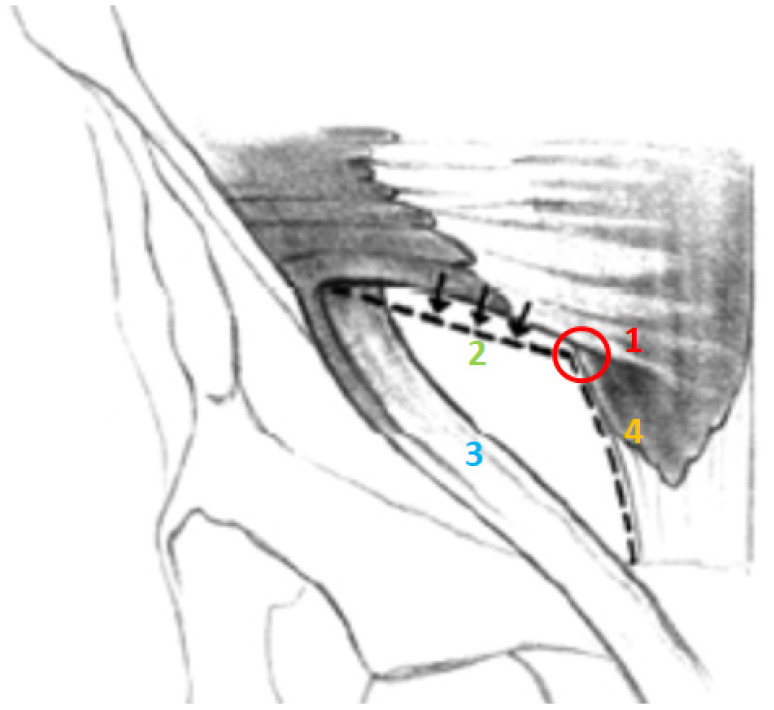
Hessert’s triangle is an anatomical area delimited by the internal ring at the apex (1), the internal oblique and the transversus abdominis muscles (2) and the inguinal ligament (3) laterally, and, at its base, by the rim of the rectus abdominis muscle (4).

The inguinal canal (IC) is another important anatomical structure of the pelvis as it channels structures from the abdominal wall to the external genitalia; it is traversed in males by the spermatic cord and, in females, by the round ligament. The IC is delimited by four walls (anterior, inferior, superior, and posterior) and presents two openings: the deep/internal inguinal ring and the superficial/external inguinal ring [36]. The anterior wall is formed principally by the aponeurosis of the external oblique muscle. The posterior wall is formed by the transversalis fascia, which is strengthened laterally by the interfoveolar ligament of Hesselbach and medially by the ligament of Henle, the ligament of Colles, and the conjoint tendon [15]. The superior wall, or the roof of the inguinal canal, is delimited by the lower rim of the internal oblique muscle and the transversus muscle, whereas the floor of the inguinal canal, or the inferior wall, is defined by the inguinal ligament, strengthened medially by the lacunar ligament. The superficial inguinal ring is a triangular-shaped opening delimited by the fibers of the external oblique aponeurosis, which originate from the anterior superior iliac spine [44]. The fibers leading up to the pubic tubercle, along which the spermatic cord or the round ligament pass, form the inferior crus (infero-lateral pillar or external pillar), whereas the fibers leading up to the pubic symphysis form the superior crus (supero-medial pillar or internal pillar) [15]. The deep inguinal ring faces the abdominal cavity and is perpendicular to the middle part of the inguinal ligament, and it lies about 15–20 mm from the inguinal ligament and about 50 mm from the pubic tubercle [15,36]. Figure 3 presents a schematic view of the inguinal canal. The inguinal region lends itself to inguinal hernia (IH) formation as it is a region of weakness in the abdominal wall. Inguinal herniations are reported in the literature as being less frequent in women than in men [37], with ratios of IH men: IH women ranging from 12:1 [24,25] to 9:1 [26]. Another gender difference regards the incidence of direct and indirect IH. Indeed, several studies report that direct hernias are very rare in the female population yet common in men [24,25,26,37,48]. Several anatomical differences between the two sexes are worth pointing out with regard to these data. Since the round ligament is narrower than the spermatic cord, in women, both the superficial [16] and the deep inguinal [17] rings are narrower in comparison to those of men, although it is important to remember that the diameter of both these rings shows a significant variability among the members of both sexes [48]. A second anatomical difference between the two sexes is that the transversalis fascia, i.e., the anatomical structure forming the posterior wall of the inguinal canal, is generally stronger in women than in men [16]. Yet another difference is the angle between the inguinal ligament and Cooper’s ligament: some authors report this angle to be smaller in women than in men [25]. This exact anatomical detail is worthy of note and could represent an important protective factor in women against IH onset. Indeed, other authors showed that if the conjoint tendon and the inguinal ligament are far apart, i.e., if the angle between the inguinal ligament and Cooper’s ligament is wide, this may constitute a risk factor for IH [38]. However, this aspect is relatively difficult to assess because there does not yet exist an accepted reference distance [38]. Conversely, the femoral hernia, another important cause of GPS [3,8], occurs approximately four times more commonly in women than in men [44]. These differences may be explained both by the wider shape of the pelvis and by the wider rectus abdominis muscle in women [44]. One final difference, of no lesser importance, regarding the inguinal anatomy of the two sexes is the width of Hessert’s triangle [18]. Hessert’s triangle is an anatomic area, triangular in shape, where the deep inguinal ring represents the apex, the internal oblique and the transversus abdominis muscles together with the inguinal ligament are the sides, and the edge of the rectus abdominis muscle is the base (Figure 4) [18]. Located within this triangle is the transversalis fascia, which is the weak point of the inguinal canal as it is not reinforced by muscle [49]. This area of weakness is closed and, therefore, protected by a physiological procedure called the “inguinal shutter” mechanism [39], during which the internal oblique and transversus abdominis muscles and their respective aponeurosis approach the inguinal ligament during contraction [40,41]. In this manner, the transversalis fascia is protected against the formation of a direct inguinal hernia [39,40,41]. When the intersection of the internal oblique and transversus abdominis muscle with the rectus abdominis sheath is bigger than normal, Hessert’s triangle becomes wider, and the closure carried out by the “inguinal shutter” mechanism may be incomplete and ineffective [19]. Since Hessert’s triangle is significantly larger in males than in females [49] this may favour the risk of developing direct IH in males [49].

## 5. Pelvic Anatomy

**Figure 5:**
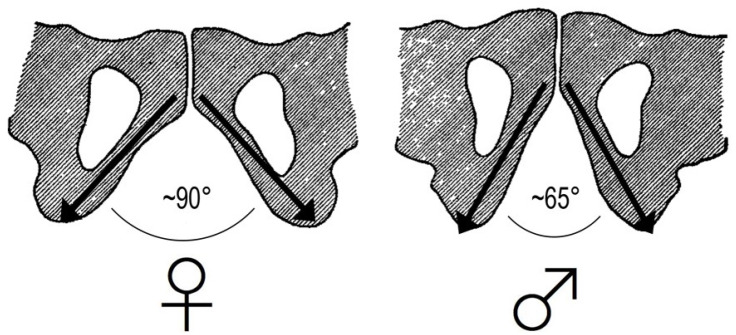
The angle between the inferior pubic rami is greater in women than in men. This difference changes in the frontal plane the force vectors of the adductor muscles (black arrows).

The pelvis contains organs of great importance such as the bladder, rectum, and sigmoid colon, common to both men and women, as well as the reproductive organs. In women, the pelvis houses the uterus, fallopian tubes, ovaries and vagina. In men, the pelvis houses the prostate, vas deferens, and seminal vesicles. In men, the pelvis develops more in height, assuming a more vertical position compared to a woman’s pelvis, which develops more in width and assumes a more forward-inclined position. In particular, the true (o lesser) pelvis is wider in women, and the pelvic inlet shows a greater medio-lateral diameter in comparison to that in men [20]. Furthermore, the angle between the inferior pubic rami is greater in women than in men (90° vs. 65°) [57] (Figure 5), and consequently, the frontal angle measurable between the midline of the body and the force vectors of the adductor muscles should theoretically be greater in females than in males. This anatomical aspect, according to several authors, may be a protective factor against traumatic lesions and overuse of the adductor muscle-tendon complex [57].

## 6. Hip Joint Anatomy

Femoral acetabular impingement (FAI) is a clinical condition characterized by an abnormal contact between the femur head–neck junction and the acetabulum [21,27,46]. Several forms of FAI have been recognized, all of which are important causes of GPS [45]: pincer-FAI, cam-FAI, and mixed forms. Pincer-FAI results from an over-coverage of the femoral head by the acetabulum, generating an abnormal contact between the femoral neck and the acetabular rim; this form seems to be more common in women than in men [21,27,46]. In cam morphology, there is an abnormal morphology of the head–neck junction with an osseous apposition over the femoral head, which forms a bony protuberance obliterating the normal head–neck offset [21,27,46]. In 62–87% of FAI cases, cam and pincer morphology may be combined to give a mixed form [22,42,51]. In athletes, the mixed form is more common in males (62%) than in females (38%) [28]. Cam-FAI is also more prevalent in men than in women [53]. Upon clinical presentation, female patients affected by FAI show a greater disability and a worse score, in comparison to male patients, in the Harris hip score (mHHS), the Western Ontario and McMaster Universities Osteoarthritis Index (WOMAC), the Hip Disability and Osteoarthritis Outcome Score (HOOS), and the SF-12 (12-Item Short Form Health Survey) physical function sub-score (all *p* ≤ 0.02) [28]. Furthermore, female patients display greater hip motion (flexion and internal rotation and external rotation in 90° of flexion; all *p* ≤ 0.003) and less severe cam-type morphologies (a mean maximum alpha angle of 57.6° compared with 70.8° for males; *p* < 0.001) [28]. Finally, males are significantly more likely to have advanced acetabular cartilage lesions (56% of males compared with 24% of females; *p* = 0.001) and larger labral tears with a more pronounced posterior extension of these abnormalities (*p* < 0.02) [28]. Some studies also show that female populations have a smaller center edge angle (i.e., a poor acetabular coverage) and a larger acetabular inclination than males. These data suggest that hip dysplasia could be more frequent in females than in males [50].

## 7. Sexual Apparatus

Differences in the sexual apparatus of the two sexes lead to a very different etiopathogenesis of GPS in men and women. Table 2 shows various inflammatory and non-inflammatory clinical conditions of the reproductive organs that can cause GPS.

## 8. Discussion

GPS is a relatively common condition affecting professional and amateur athletes as well as the athletic “weekend warriors” [52]. It is a particular problem in soccer, football, ice hockey, handball, tennis, and rugby [2], where cutting maneuvers, changes in direction, and kicking are all part of the action. Furthermore, several studies demonstrate an important gender-based difference in the incidence of GPS in both sexes, albeit attributing different ratios of female:male GPS incidence: Hynes et al. [9] report a 1:9.2 ratio, another study [5] provides an even greater ratio of 1:19.2, a ratio of 1:7.8 emerged from two other recent studies [7,8] and one rather recent review [6] shows that for the same sport practiced at the same level, men are 2.5 times more likely to suffer from GPS than women (relative risk, RR 2.45, 95% CI 2.06 to 2.92).

These data can, in actual fact, be partially explained by the anatomical differences that exist between the two sexes. 

An important cause of GPS is PPAC injury due to trauma or functional overload [35]. Interestingly, it transpires that PPAC anatomy is different in men and women [14]: direct insertion of the rectus abdominis muscle onto the antero-superior part of the pubic symphysis in women could act as a protective factor against PPAC lesions [14] whereas in males, the greater recto-gracilis tendinous extension could expose them to traumatic and/or overuse lesions of the PPAC portion afferent to the recto-gracilis junction. These hypotheses need to be supported by valid epidemiological studies, which, to the best of our knowledge, are not yet present in the current literature.

IH is another important cause of GPS in athletic populations [3,4,7,8], where men are 9–12 times more susceptible to this type of onset than women [44]. This observation may also have a gender-based anatomical explanation. Indeed, several studies show that the frequency of indirect hernias is two times higher in men than in women [37]. In fact, the deep inguinal ring in males tends to have a larger transverse diameter than that of females [17], and this wider, deep inguinal ring in men could lead to a less effective inguinal shutter mechanism, already described by Lytle, and consequently to the onset of indirect IH and ultimately to GPS. Furthermore, the significantly greater width of Hessert’s triangle in men may represent a risk factor for the onset of direct IH [49]. Moreover, several other anatomical differences present in women may, at least in theory, safeguard them against the onset of IH [16,25,38]. Compared to male anatomy, these differences include a stronger transversalis fascia, a narrower superficial ring, and a closer angle between the inguinal ligament and Cooper’s ligament. Yet, several studies in the literature do not report any statistical difference in the incidence of IH between sporting populations of the two sexes [8,16,23,50]. One reason for this may be a missed diagnosis of IH in the female population due to a gender-related anatomical difference that makes exploration of the superficial inguinal ring more difficult in females than in males [29]. Furthermore, Spangen et al. [16] described inguinal canal posterior wall weakness (ICPWW) in a subgroup of female patients as a clinical situation in which a persistent groin- and lower abdominal pain is present without the presence of a palpable hernia. Some authors [8,16] underlined that when present, ICPWW is amplified by physical activity and, during the execution of the Valsalva maneuver, is always accompanied by a distinctive, specific soreness located precisely above the internal inguinal ring. This exact clinical condition is referred to as an “occult hernia” by other authors [30,58] and may very well be an unnoticed cause of GPS in females [8,30,58].

One particularly interesting aspect that could account for GPS not being attributed to IH in female athletes is the existence of a bias, unconsciously held by the female athletes themselves [9], regarding patient compliance. Indeed, a gender-linked disparity toward treatment has already been demonstrated in a variety of clinical conditions. For example, despite osteoarthritis being more frequent in females, female patients are less likely to undergo joint arthroplasty than men [59,60]. Furthermore, women are also more reluctant to undergo imaging tests for chronic wrist pain [61]. The same type of bias could be present in the case of GPS caused by IH. Therefore, the possibility of LSGPS being caused by an IH in female patients should be taken into account and not overlooked.

Several studies of female athletic populations [7,8] confirm that femoral hernias are more frequent in women [44] because of their wider pelvis and wider rectus abdominis muscle [44]. Finally, it is interesting to note that, with respect to their male counterparts, female athletes are more prone to suffering from chronic pain after hernia surgery [47].

The anatomical difference between the pelvis of the two sexes also has to be considered when analyzing GPS etiopathogenesis. Females tend to have a wider pelvis, which confers a more oblique angle of action for the short adductors muscle (gracilis, adductor brevis, and pectineus muscles), which, in turn, may reduce the traction force exerted at the proximal origin of these muscles [31]. Some authors consider this anatomical aspect [57] to be a protective factor against overuse and traumatic injuries of the short adductor muscle-tendon complex. Although this hypothesis may well have found confirmation in a study by Hynes et al. [9] in which female athletes prove to be significantly less likely to incur short adductors injuries (RR = 0.14; *p* < 0.005), additional, valid epidemiological supporting studies still need to be carried out.

One of the most important clinical conditions causing GPS is FAI [45]. Again, there are gender-based traits to the nature of the underlying form of FAI: women present a greater disposition toward contracting pincer-FAI than men [21,27,46], while cam-FAI and the mixed forms are more common in males [28,53]. The high incidence of hip dysplasia [50,56] and the greater occurrence of pincer-FAI in females [8,27,46] could explain the greater predisposition, especially in female athletes, toward developing acetabular labral injury compared to their male counterparts. This hypothesis is supported by several studies that report a greater occurrence of acetabular lesions in women as a cause of LSGPS [7,8]. On the other hand, the higher incidence of cam morphology in the male population has another basis. During skeletal maturation of the hip joint, the femoral growth plates remain open [54,55] and are susceptible to the effects of high-impact sports practiced assiduously during this phase of development: as a result, cam-morphology FAI evolves. Since it is proven that during early adolescence, boys are more physically active than girls [62,63] and typically tend to participate in such high-impact sports as soccer, football, basketball, and hockey, this could explain why the incidence of cam morphology FAI in males is higher than that seen in females.

Differences in the sexual apparatus of the two sexes lead to a very different etiopathogenesis of GPS in men and women, as shown in Table 2. Varicocele in men [7] and endometriosis in females [8] are the disorders most commonly associated with the onset of GPS, as several studies report. In addition to the anatomical differences between the sexes, the greater incidence of GPS in males can be explained by the greater physical activity carried out by men in general, and particularly by their greater participation in high-impact sports compared to women [50,62,63] and by the particular intensity of training loads and/or the match workloads [5,6,7,8,9] that male athletes engage in compared to female athletes.

## 9. Conclusions

Although an increasing problem in many sports, GPS displays a greater incidence in males than in females on the whole. There are, however, various considerations to be made. Whereas the female anatomy seems to be innately protective against several clinical conditions such as IH and adductor muscle injuries, in the case of hip pathologies, the conditions that consequently give rise to GPS seem to be different in the two sexes. In addition, the greater physical activity of males in general and the differential participation of the two sexes in high-impact sports could, at least in part, explain the gender difference in the incidence of GPS.

Further sex-specific studies must be carried out to better understand how the differences in male and female anatomy can influence, to different extents, the onset of GPS.

## 10. Future Direction

Several aspects still need to be clarified; in particular, the inconsistency between the low theoretical incidence of IH in female athletes and the results of several recent studies indicate the contrary. Thus, more evidence-based epidemiological studies on women who practice sports associated with a high risk of developing GPS are needed.

## Figures and Tables

**Table 2:** Sexual apparatus disease-related causes (inflammatory and non-inflammatory) causing GPS in men (column 1) and women (column 2) [3,4].

Men	Women
Prostatitis	Ovarian cysts
Epididymitis	Endometriosis
Corditis	Ectopic pregnancy
Orchitis	Round ligament entrapment
Varicocele	Ovarian torsion
Hydrocele	Other infections of the urinary tract
Cystitis	
Urethritis	
Testicular torsion	
Other infections of the urinary tract	

## Data Availability

Not applicable.

## Abbreviations

**Abbreviation**	**Full Text**
GPS	Groin pain syndrome
LSGPS	Long-standing groin pain syndrome
PPAC	Prepubic aponeurotic complex
SPL	Superior pubic ligament
IPL	Inferior pubic ligament
APL	Anterior pubic ligament
PPL	Posterior pubic ligament
IC	Inguinal canal
IH	Inguinal hernia
FAI	Femoral acetabular impingement
ICPWW	Inguinal canal posterior wall weakness
